# Clinical profiles and molecular genetic analyses of 98 Chinese children with short statures

**DOI:** 10.3389/fgene.2024.1364441

**Published:** 2024-06-12

**Authors:** Danfeng Fang, Xing Li, Zhigang Zhang, Hefei Cai, Lu Wang, Jiahe Yu, Xuanye Hu, Bin Ye

**Affiliations:** ^1^ Taizhou Central Hospital (Taizhou University Hospital), Taizhou, China; ^2^ Department of Pediatric Endocrinology/Genetics, Xinhua Hospital, School of Medicine, Shanghai Jiao Tong University, Shanghai, China

**Keywords:** short stature, phenotype, genetic etiology, whole-exome sequencing, recombinant human growth hormone treatment

## Abstract

**Background:**

Short stature is one of the most prevalent endocrine disorders in children, and its genetic basis is a complex and actively researched subject. Currently, there is limited genetic research on exome sequencing for short stature, and more large-scale studies are necessary for further exploration.

**Methods:**

The retrospective study entailed investigation of 98 Chinese children with short statures (height SDS ≤ −2.5) of unknown etiologies recruited between 2017 and 2021. Whole-exome sequencing (WES) was performed on these patients to identify the potential genetic etiologies. The clinical data were reviewed retrospectively to assess the pathogenicity of the identified mutations. Additionally, 31 patients consented to and received recombinant human growth hormone (rhGH) therapy for 12 months. The short-term effects of rhGH treatment were evaluated across different etiologies of patients with short statures.

**Results:**

The WES results were used to identify 31 different variants in 18 genes among 24 (24.5%) patients. Individuals with more severe short statures were more likely to have underlying genetic etiologies. Short stature accompanied by other phenotypes had significantly higher diagnostic yields than simple severe short stature. The rhGH therapy demonstrated efficacy in most children. Nevertheless, the treatment response was suboptimal in a boy diagnosed with 3M syndrome.

**Conclusion:**

WES is an important approach for confirming genetic disorders in patients with severe short statures of unknown etiologies, suggesting that it could be used as a primary diagnostic strategy. The administration of rhGH may not be suitable for all children with short statures, and the identification of the genetic cause of short stature by WES has significant guidance value for rhGH treatment.

## Introduction

Short stature is characterized by a height (or length) that falls two standard deviations (-2SD) below the average for individuals among the same race, region, age, and gender under a similar growth context; it ranks among the most prevalent pediatric endocrine disease phenotypes (incidence = 2%) (Cohen, 2014). Children with severely short statures often experience mental health challenges as well as diverse developmental, educational, and social issues (Siegel et al., 1991). The process of growth is complex and influenced by various factors, including heredity, hormones, nutrition, and the environment. While hereditary factors play a major role in determining individual heights, accounting for 80% of the variation, a significant portion of these genetic factors (estimated at 60%–80%) remains unidentified (Pedicelli et al., 2009). In recent years, genome-wide association analysis (genome-wide association study, GWAS) has provided insights into the genetic basis of height. In 2014, the largest US Genetic Study of humans (Genetic Investigation of Anthropometric Traits, GIANT) was conducted with 253,288 samples, from which researchers identified 697 height-related single-nucleotide polymorphisms (SNPs) in 423 gene regions. The cumulative effect of common variants in these microactive genes can explain 20% of the variation in the height of the population (Wood et al., 2009). However, pathogenic mutations in monogenic genes can significantly impact height (Dauber et al., 2014). Advancements in molecular technologies have led to the development and application of various genetic testing techniques to study the etiologies of short stature. While the classical Sanger sequencing is both time- and labor-intensive, whole-exome sequencing (WES) has shown a high diagnostic yield for patients with short statures (Guo et al., 2014; Hauer et al., 2018). However, the data and studies based on exome sequencing for genetic evaluation of short stature are limited and constrained by regional and ethnic factors, necessitating further large-scale investigations (Huang et al., 2018; Li et al., 2022). The massive data analysis and report interpretation by WES present new challenges to scientific research applications and clinical practice.

The introduction of recombinant human growth hormone (rhGH) therapy in 1985 offered hope for treating children with short statures. However, the effectiveness of rhGH treatment varies significantly among individuals, as noted through long-term clinical observations and analyses of extensive clinical data both domestically and internationally. Although rhGH therapy has been recommended for patients with certain syndromes, such as Turner syndrome, Prader–Willi syndrome, and Noonan syndrome (Noonan and Kappelgaard, 2015; Butler et al., 2019; Gravholt et al., 2019), its efficacy remains uncertain for many other syndromic short statures. Therefore, in the process of exploring the genetic etiologies of short stature in children, it is crucial to investigate whether there exist genetic variants associated with the efficacy of rhGH treatment and to evaluate the roles of different pathogenic genes in rhGH treatment sensitivity. These findings will then serve as a foundation for personalized treatment based on the genotype, facilitating precision medicine in the future.

In this study, we collected samples from 98 Chinese patients with a height SDS ≤ −2.5 and unknown etiologies for analyses using WES. We conducted a comprehensive analysis covering clinical manifestations, radiological examinations, variant interpretations, pathophysiology, and responses to rhGH treatment. This work describes the gene mutation analysis findings in patients with short statures; thus, it provides insights into the current understanding of the etiologies and responses to rhGH treatment in children with short stature.

## Materials and methods

### Patient selection and study design

**FIGURE 1:**
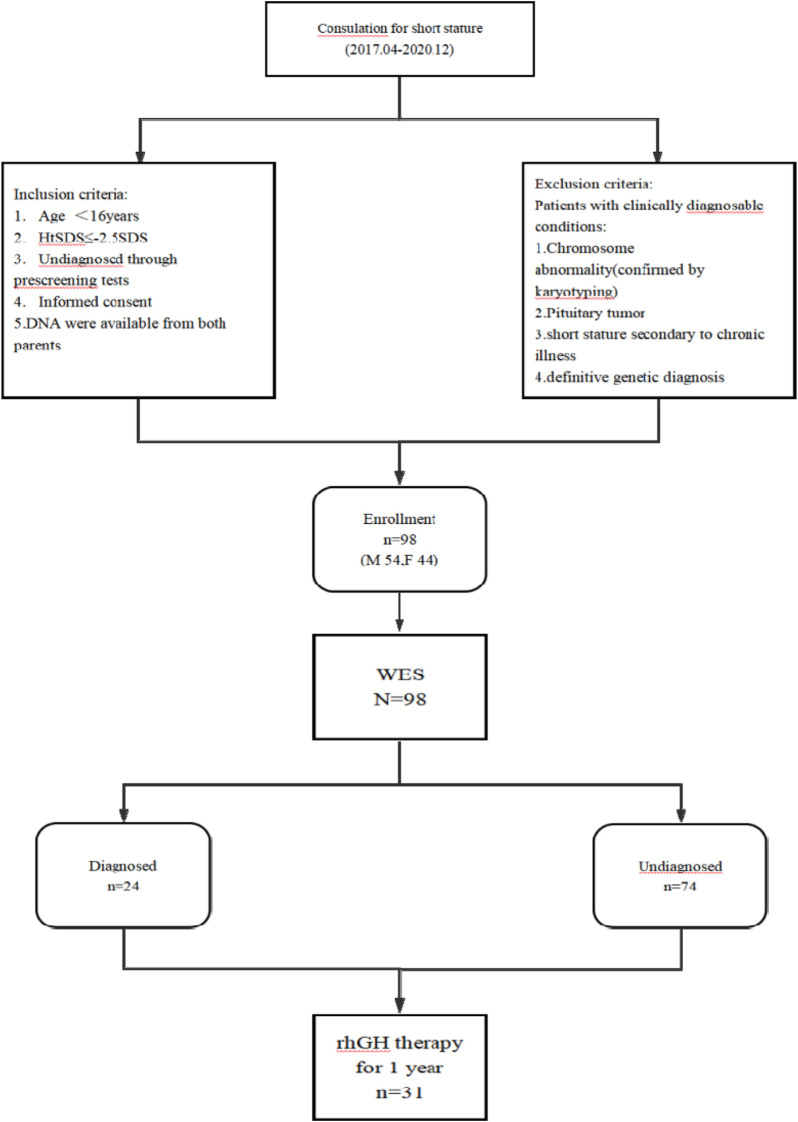
Flowchart of study subjects. Note: M, male; F, female; n, number; HtSDS, standard deviation score of height; WES, whole-exome sequencing.

From April 2017 to December 2021, a total of 98 children with short stature who were being followed up at Taizhou Central Hospital (Taizhou University Hospital) were enrolled in our cohort for pathogenic screening. The inclusion criteria were as follows: age below 16 years; body height below −2.5 standard deviation units (−2.5 SDS) compared to age- and sex-matched Chinese population (Li et al., 2009); undiagnosed cause through prescreening tests. The exclusion criteria included the following: chromosome abnormality confirmed by karyotyping; pituitary tumor; short stature secondary to chronic illness; definitive genetic diagnosis (Figure 1). The Ethics Committee of Taizhou Central Hospital approved this study (2023L-08-06), and written informed consent was obtained from each of the participants or their guardians before performing DNA isolation.

The documented medical histories of the 98 patients included their birth status, feeding, growth, and history of illness as well as those of their family members. Physical examinations were conducted for the facial features, body height, weight, and head circumference at the first visit/referral or before rhGH treatment. Growth hormone (GH) provocation tests were then performed via two independent provocation tests (arginine and clonidine). Partial GH deficiency (PGHD) was confirmed for serum peak GH levels of 5–10 ng/mL, and complete GHD (CGHD) was confirmed for serum peak GH levels of < 5 ng/mL. Another laboratory-based evaluation involved the level of the insulin-like growth factor (IGF-1), where a value lower than -2.0 standard deviation units with respect to sex- and age-matched references was defined as IGF-1 deficiency (Juul et al., 1995; Xu et al., 2010). The bone age (BA) was assessed by radiographic imaging and read according to the Greulich–Pyle Atlas method. Most patients were also assessed on the basis of pituitary magnetic resonance imaging (MRI) and echocardiography. Any developmental delay/intellectual disability (DD/ID) was confirmed using the Gesell Developmental Scale or Wechsler Intelligence Scale.

### Molecular genetic analysis

Genomic DNA samples were isolated from the peripheral blood of the 98 patients and their parents using blood genomic DNA extraction kits (Zeesan Biotech, China), and the capture library was prepared using SureSelect All Exon V5 (Agilent). The library was sequenced using the Illumina HiSeq 4,000. The operations were performed in accordance with the standard operating procedures, including genomic DNA fragmentation processing, end repair, adapter ligation, amplification, probe hybridization target region, and capture enrichment by the magnetic bead method, among others. Raw data were obtained in the FASTQ format using the software provided by the sequencing platform. These raw data were then aligned to the human reference hg19 using the Burrows–Wheeler alignment (BWA) tool. The overrepeated detected data were denoised and evaluated for quality using Picard tools. Furthermore, the single-base, insertion, and deletion variants were identified using the Genome Analysis Toolkit (GATK). The output vcf files were next annotated using SNPEff, and the high-frequency variants were filtered for allele frequency (MAF) of >0.01 in genomAD, 1000 Genomes Project, and Exome Aggregation Consortium (ExAC). The filtered variants were subsequently sorted on the basis of the inherited patterns and correlations between the patient phenotypes and mutant genes. The candidate variants were then classified according to the guidelines of the American College of Medical Genetics and Genomics (ACMG) after comprehensive analysis of all results (Richards et al., 2015). Ultimately, all the identified variants were confirmed and validated by Sanger sequencing.

### GH treatment

After obtaining informed consent from the parents of some participants and excluding any contraindications, rhGH treatment was initiated to improve height. Based on the etiologies, the patients were divided into five groups as the PGHD, CGHD, idiopathic short stature (ISS), syndromic short stature (SSS), and genetic skeletal disorder (GSD) groups. During the course of rhGH therapy, the heights and weights of the patients were measured and recorded every 3 months, and the body mass index (BMI) values were calculated. Some biochemical parameters were also monitored every 3 months, such as blood glucose, liver function, kidney function, insulin (INS), thyroid function, and IGF-1. The BA was examined every 6 months to 1 year. Notably, we identified some adverse effects of rhGH therapy, such as headaches, impaired glucose metabolism, liver damage, hypertension, arthralgia, and tumors. The rhGH dosage was then adjusted according to the growth velocity and IGF-1. The data for the first year of GH treatment were collected, and the short-term effects of rhGH on short patients with different etiologies were analyzed by comparing the relevant data before and after treatment, including the growth velocity, standard deviation of height, standard deviation of BMI, standard deviation of IGF-1, and difference between BA and actual age.

### Statistical analyses

All statistical analyses were performed using SPSS software version 27.0. The Pearson’s χ2 test, Yates correction for continuity, and Fisher’s exact test were performed for differences in the diagnostic categories between groups, and results with *p* < 0.05 were considered to be statistically significant. The odds ratio (OR) and 95% confidence interval (CI) were also calculated for each diagnostic category. Furthermore, forest plots were generated using the Stata 13.0 statistical package, and ANOVA was used to compare the growth data of rhGH therapy among the five study groups.

## Results

### General data

This study included 98 participants [including 54 male (55.1%) and 44 female (44.9%)] with an average age of 7.37 ± 3.06 years (range: 1.42–14.33 years). The standard deviation score of the average height (HtSDS) of the patients was −3.1 ± 0.61 (range: 2.52 to −5.61). Table 1 summarizes the clinical characteristics of the participants in this study.

**TABLE 1:** Diagnostic yields of the 98 patients with short statures and different phenotypes.

	*n* (%) (Total = 98)	Diagnostic yield	*p*-Value
Family			0.644
Yes	29 (29.6%)	8 (27.6%)	
No	69 (70.4%)	16 (23.2%)	
GH provocation			
Deficiency	60 (61.2%)	17 (28.3%)	
CGHD	21 (21.4%)	9 (42.9%)	
Sufficiency	35 (35.7%)	6 (16.7%)	
NA	2 (2.0%)	—	
IGF-1			0.948
Deficiency	59 (60.2%)	14 (23.7%)	
Sufficiency	26 (26.5%)	6 (23.1%)	
NA	13 (13.3%)	—	
Bone age			0.209
Delayed	71 (72.4%)	15 (21.1%)	
Normal	27 (27.6%)	9 (33.3%)	
Pituitarium MRI			1.000
Abnormal	6 (6.1%)	2 (33.3%)	
Normal	71 (72.5%)	21 (29.6%)	
NA	21 (21.4%)	—	
Short stature with additional phenotypes	33 (33.7%)	23 (69.7%)	
Short stature with one additional phenotype	13 (13.3%)	7 (53.8%)	
Short stature with more than one additional phenotype	20 (20.4%)	16 (80.0%)	
Main additional phenotypes			
SGA	4 (4.1%)	0 (0.00%)	—
Microcephaly/macrocephaly	6 (6.1%)	5 (83.3%)	0.003
Facial dysmorphism	19 (19.4%)	16 (84.2%)	<0.001
Skeletal abnormalities	12 (12.2%)	10 (83.3%)	<0.001
DD/ID	11 (11.2)	9 (81.8%)	<0.001
Cardiac anomaly	6 (6.1%)	4 (66.7%)	0.047

### Genetic variants identified

In total, samples from 98 participants with suspected monogenic short stature were assessed by WES. The final diagnostic yield was 24.5% (24/98) for all patients. We identified 31 variants in 18 genes, including nine variants that have not been reported previously (Table 2, detailed in Supplementary Table S1; Table 2). According to the ACMG guidelines, most variants were identified as pathogenic or likely pathogenic. However, two of the variants were classified with uncertain significance; they were considered a possible genetic etiology owing to the patient’s phenotype, and the inheritance patterns coincided with those of the corresponding genetic disorders.

**TABLE 2:** WES findings associated with a molecular diagnosis.

Gene	Associated disease	Inheritance	Sequencing variants (hg19)^a^	Classification^b^	Patient
Autosomal dominant					
*CREBBP*	Rubinstein-Taybi syndrome	*De novo*	NM_004380.2:c.6111del(p.Arg2037SerfsTer3)	LP	P9
*COL1A2*	Osteogenesis imperfecta, type IV	*De novo*	NM_000089.3:c.3256C>T(p.Gln1086*)	P	P23
*KRAS*	Noonan syndrome 3	*De novo*	NM_004985.4:c.40G>A(p.Val14Ile)	P	P38
*NF1*	Neurofibromatosis-Noonan syndrome	*De novo*	NM_000267.3:c.60G>C(p. Gln20His)	LP	P40
		Maternal	NM_000267.3:c.943C>T(p.Gln315Ter)	P	P81
	Neurofibromatosis, type 1	*De novo*	NM-001042492.2:c.693delT(p.Phe231fs)	P	P57
*KMT2A*	Wiedemann-Steiner syndrome	*De novo*	NM_001197104.1:c.3460C>T(p.Arg1154Trp)	P	P62
*GH1*	Growth hormone deficiency, isolated, type II	*De novo*	NM-000515.4:c.626G>A(p.Arg209His)	LP	P65
*COMP*	Pseudoachondroplasia	Maternal	NM-000095.2:c.1829A>G(p.Tyr610Cys)	LP	P66
*FGFR3*	Achondroplasia	*De novo*	NM_000142.4:c.1138G>A(p.Gly380Arg)	P	P79
	Hypochondroplasia	Paternal	NM_000142.4:c.1619A>G(p.Asn540Ser)	P	P96
		*De novo*	NM_000142.4:c.1620C>G(p.Asn540Lys)	P	P97
*FBN1*	Acromicric dysplasia	Paternal	NM_000138.4:c.5183C>T(p.Ala1728Val)	LP	P84
*BMP4*	Microphthalmia, syndromic 6	*De novo*	NM_001202.6:c.371-2A>G	LP	P85
*ARID1B*	Coffin-Siris syndrome	*De novo*	NM_020732.3:c.6683C>A(p.Ser2228*)	LP	P86
		*De novo*	NM_020732.3:c.4520delA(p.Asn1507fs)	P	P98
Autosomal recessive					
*FLNB*	Spondylocarpotarsal Synostosis syndrome	Maternal	NM_001457.3:c.1945C>T(p.Arg649Ter)	P	P4
		Paternal	NM_001457.3:c.2774G>A(p.Gly925Asp)	LP	
		Maternal	NM_001457.3:c.2452C>T(p.Arg818Ter)	P	P17
		Paternal	NM_001457.3:c.4819C>T(p.Arg1607Ter)	P	
*SLC12A3*	Gitelman syndrome	Paternal	NM_000339.2:c.179C>T(p.Thr60Met)	P	P7
		Maternal	NM_000339.2:c.1316G>T( p.Gly439Val)	LP	
*ALPL*	Hypophosphatasia, infantile	Maternal	NM_000478.4:c.212G>A(p.Arg71His)	P	P8
		Paternal	NM_000478.4: c.571G>A(Glu191Lys)	LP	
*C5orf42*	Orofaciodigtal syndrome	Paternal	NM_023073.3:c.3577C>T (p.Arg1193Cys)	LP	P11
		Maternal	NM_023073.3:c.3599C>T (p.Ala1200Val)	P	
*CUL7*	3M syndrome 1	Paternal	NM_014780.4:c.4318C>T(p.Arg1440*)	P	P13
		Maternal	NM_014780.4:c.2875dupA(p.Thr959fs)	P	
*HGSNAT*	Mucopolysaccharidosis type ⅢC	Paternal	NM_152419.2:c.1516C>T(p.Arg506Ter)	P	P16
		Maternal	homozygous		
*KIAA0196*	Ritscher-Schinzel syndrome	Paternal	NM_014846.3:c.232C>T(p.Gln78Ter)	P	P30
		Maternal	NM_014846.3:c.2489G>A(p.Arg830Gln)	LP	

^a^ Novel variants (i.e. variants absent in population and disease databases) are labeled underlined.

^b^ According to the recently published ACMG standards and guidelines.

Abbreviations: P, pathogenic; LP, likely pathogenic.

Among all the mutant genes, there were 11 that were related to autosomal dominant disorders. The majority of the identified variants manifested *de novo*, except for the variants carried by P66 and P81 that were maternally inherited and those of P84 and P96 that were paternally inherited. The other affected genes followed an autosomal recessive pattern of inheritance. With the exception of the variants carried by P16 that were homozygous, all other variants were compound heterozygous. Furthermore, all variants were identified to have paternal and maternal origins by Sanger sequencing.

Additionally, it was found that at least 12 genes (12/18) affect the recognized growth plate regulatory systems and skeletal development (Wang et al., 2013; Wit et al., 2016). However, only one case (P65) harbored mutations in the GH–IGF1 axis; this indicates that clinicians should focus on the growth plate and skeletal development pathway.

### Diagnostic yield of WES

**FIGURE 2:**
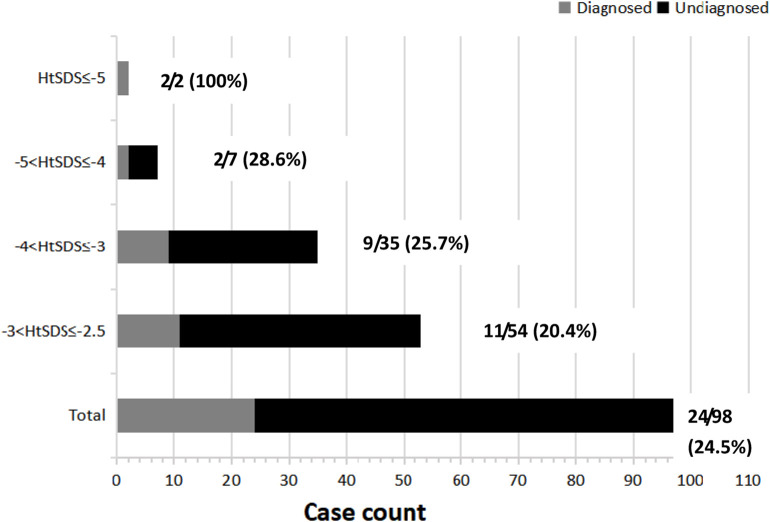
HtSDS distribution and corresponding diagnostic yields. Next to each bar, the denominator represents the total number of cases (shown in gray and black rectangles), and the numerator represents the solved cases (shown in gray rectangles) corresponding to a given HtSDS range.

Based on the HtSDS range, all the patients were divided into four different groups. Compared with the overall yield (24.5%), patients with HtSDS between −2.5 and −3 had lower diagnostic rates (20.4%), and those with HtSDS between −4 and −5 as well as below −5 had higher diagnostic rates (28.6% and 100%, respectively). This suggests that patients with more severe short statures have higher probabilities of genetic etiology. However, no significant differences were found among the HtSDS groups (Figure 2, *p* = 0.111).

Sixty patients were diagnosed with GHD based on the clinical, laboratory, and imaging results, and variants were detected in 17 (28.3%) out of 60 patients. Moreover, patients with CGHD had higher diagnostic rates (9/21, 20.4%). Fourteen out of 59 patients having IGF-1 deficiency had the variants (23.7%). Four patients diagnosed with SGA did not have a genetic cause. Thirty-three patients had at least one additional phenotype; the diagnostic yields of patients with additional phenotypes were higher than the overall yield (23/33, 69.7%), and patients with more than one additional phenotype had higher diagnostic rates than those with only one additional phenotype (7/13, 53.8% *vs* 16/20, 80.0%; Table 1). However, we found no significant difference between the two subgroups (χ^2^ = 1.464, *p* = 0.226).

**FIGURE 3:**
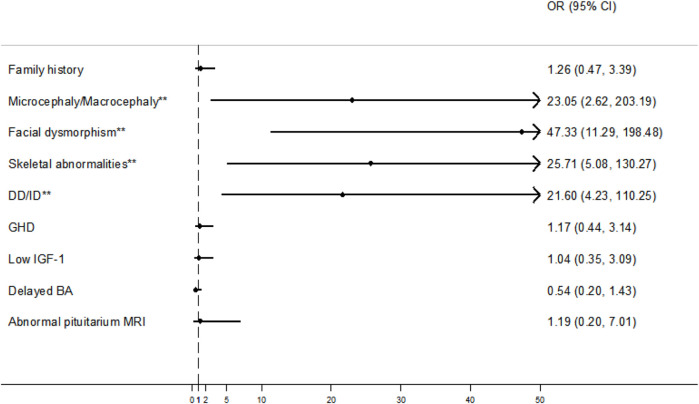
Forest plots with odds ratios (ORs) and confidence intervals (CIs) for some clinical factors. An OR with a lower 95% CI > 1 (vertical dash line) was considered a positive factor for molecular diagnosis. **p < 0.05. Note: DD/ID, developmental delay/intellectual disability; GHD, growth hormone deficiency; IGF-1, insulin-like growth factor 1; BA, bone age; MRI, magnetic resonance imaging.

Among the 30 patients with short stature and additional phenotypes, the most prevalent were microcephaly/macrocephaly, facial dysmorphism, skeletal abnormalities, and DD/ID in 6 (20.0%), 19 (63.3%), 12 (40.0%), and 11 (36.7%) cases, respectively. Furthermore, by comparing the diagnostic rates between patients with and without the additional phenotypes, we found that the aforementioned four phenotypes were statistically significant: microcephaly/macrocephaly (83.3% *vs* 20.7%, χ^2^ = 8.818, *p* = 0.003), facial dysmorphism (84.2% *vs* 10.1%, χ^2^ = 41.540, *p* < 0.001), skeletal abnormalities (83.3% *vs* 16.3%, χ^2^ = 22.107, *p* < 0.001), and DD/ID (81.8% *vs* 17.2%, χ^2^ = 18.668, *p* < 0.001) (Figure 3).

### Effectiveness during the first year of rhGH treatment

Among all the patients in this study, 31 patients (15 male and 16 female) were treated with rhGH for more than 1 year, and the average age of these patients at the start of therapy was 7.09 ± 2.85 years. The 31 patients included nine with PGHD, seven with CGHD, seven with ISS, six with SSS, and two with GSD. The median GH dose for these patients was 0.33 (0.30–0.35) mg/kg/week. During the treatment period with rhGH, all patients had neither early nor delayed puberty nor any adverse effects.

**FIGURE 4:**
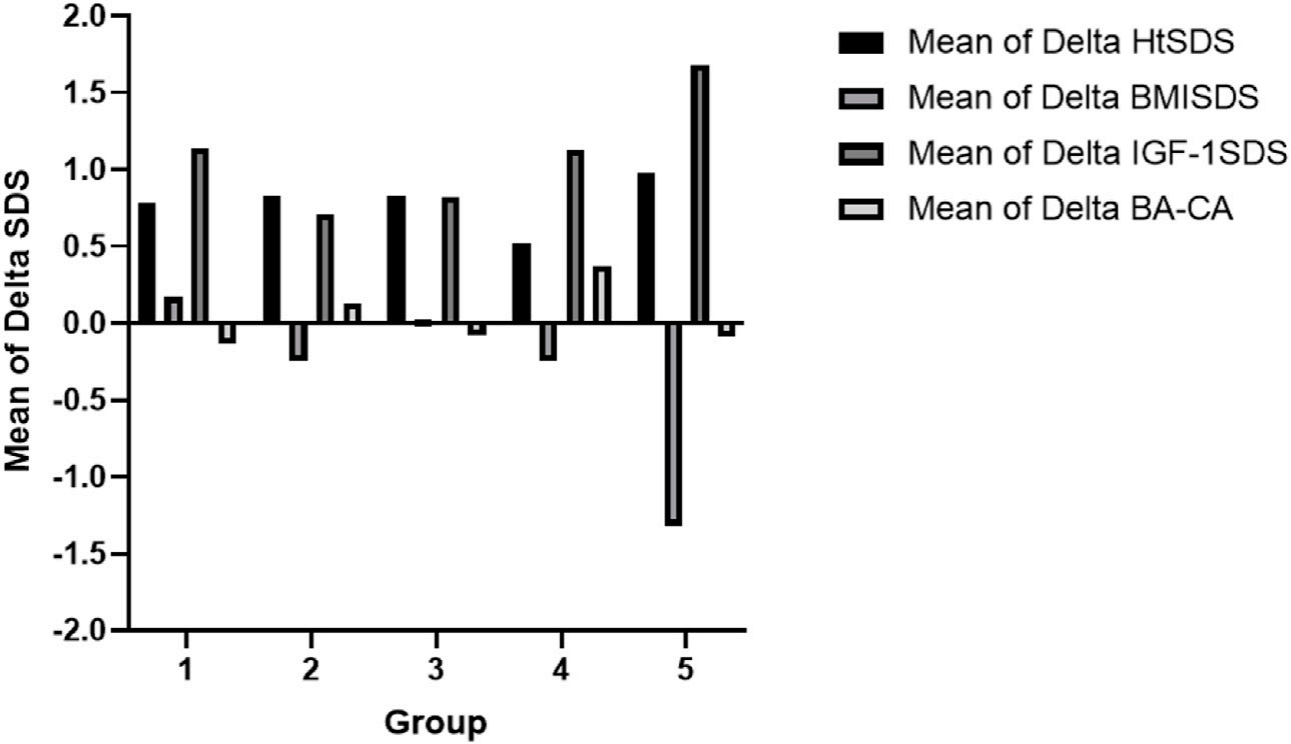
Comparison of the clinical data after treatment between the respective groups: Group 1, partial growth hormone deficiency (PGHD); Group 2, complete growth hormone deficiency (CGHD); Group 3, idiopathic short stature (ISS); Group 4, syndromic short stature (SSS); Group 5, genetic skeletal disorders (GSD). Note: HtSDS, standard deviation score of the height; BMI SDS, standard deviation of body mass index; IGF-1 SDS, standard deviation of insulin-like growth factor 1; BA, bone age; CA, chronological age.

Table 3 shows the clinical data registered from the five studied groups before rhGH treatment and after 1 year of GH therapy. Comparing the five treatment groups, the growth velocity (GV) and HtSDS showed increasing trends; ANOVA showed that there were no statistical significances between the groups (*p* > 0.05). However, the GV and HtSDS improvements in the SSS group were relatively worse than those in the other groups (Table 3; Figure 4). We analyzed the HtSDS data of the six patients in the SSS group and found that one patient with 3M syndrome (P13) had poorer height improvement than the others (GV = 7.4 cm/year, ΔHtSDS = 0.24). On the contrary, one patient with microphthalmia syndromic 6 (P85) showed better effects from therapy (GV = 10.4 cm/year, ΔHtSDS = 0.99).

**TABLE 3:** Clinical characteristics of rhGH-treated patients by the main diagnostic group. Abbreviations: PGHD, partial growth hormone deficiency; CGHD, complete growth hormone deficiency; ISS, idiopathic short stature; SSS, syndromic short stature; GSD, genetic skeletal disorders; GV, growth velocity; HtSDS, standard deviation score of height; BMI SDS, standard deviation of body mass index; IGF-1 SDS, standard deviation of insulin-like growth factor 1; BA, bone age; CA, chronological age; a, before treatment; b, after one year; F-value and *p*-value were calculated for the mean differences of the clinical characteristics between the five diagnostic groups.

	PGHD (*n* = 9)	CGHD (*n* = 7)	ISS (*n* = 7)	SSS (*n* = 6)	GSD (*n* = 2)	F-value	ANOVA (*p*-value
GV (cm/year)	9.5 ± 1.3	10.01 ± 1.23	8.96 ± 1.13	8.22 ± 1.14	9.85 ± 1.06	2.115	0.108
HtSDS^a^	−2.94 ± 0.42	−2.99 ± 0.33	−3.34 ± 0.55	−3.27 ± 1.20	−4.09 ± 0.83	1.447	0.247
HtSDS^b^	−2.16 ± 0.54	−2.16 ± 0.45	−2.51 ± 0.55	−2.76 ± 1.34	−3.11 ± 0.96	1.169	0.347
ΔHtSDS	0.78 ± 0.32	0.83 ± 0.32	0.83 ± 0.21	0.52 ± 0.27	0.98 ± 0.13	1.602	0.204
BMI SDS^a^	−0.64 ± 1.03	−0.71 ± 1.21	−0.60 ± 1.13	0.19 ± 0.73	1.47 ± 0.69	2.421	0.074
BMI SDS^b^	−0.46 ± 0.75	−0.96 ± 1.06	−0.58 ± 0.54	−0.06 ± 0.63	0.15 ± 0.07	1.553	0.216
ΔBMI SDS	0.17 ± 0.58	−0.25 ± 0.86	0.02 ± 0.75	−0.25 ± 0.58	−1.32 ± 0.62	2.069	0.114
IGF-1 SDS^a^	−1.48 ± 0.99	−1.22 ± 1.05	−1.60 ± 0.31	−1.40 ± 0.78	−1.34 ± 0.93	0.188	0.943
IGF-1 SDS^b^	−0.34 ± 1.01	−0.52 ± 1.25	−0.75 ± 0.94	−0.27 ± 1.39	0.34 ± 0.95	0.424	0.790
ΔIGF-1 SDS	1.14 ± 1.00	0.71 ± 0.59	0.82 ± 0.82	1.13 ± 1.10	1.68 ± 0.02	0.644	0.636
BA^a^-CA^a^	−2.02 ± 0.68	−1.64 ± 0.31	−1.23 ± 0.38	−1.83 ± 0.71	−2.00 ± 0.59	2.268	0.089
BA^b^-CA^b^	−2.16 ± 0.87	−1.51 ± 0.69	−1.31 ± 0.32	−1.46 ± 0.79	−2.09 ± 0.47	1.956	0.131
Δ(BA-CA)	−0.14 ± 0.32	0.13 ± 0.40	−0.08 ± 0.15	0.37 ± 0.82	−0.09 ± 0.12	1.434	0.251

All patients maintained their BMI SDS within a certain range during treatment (Figure 4). The IGF-1 SDS of all the groups increased significantly after the first year of treatment. However, the IGF-1 SDS of the ISS group increased relatively slowly (Figure 4). The differences in the IGF-1 SDS and BMI SDS among the groups were not statistically significant (*p* > 0 .05). Comparing before rhGH treatment and after 1 year of GH therapy, the differences between the BA and chronological age (CA) were not significantly different for all groups (Figure 4).

## Discussion

Growth is a complex process regulated by several genetic factors, including single-gene mutations, copy number variants (CNVs), and imprinting disorders, among others. Owing to the genetic heterogeneity and clinical complexity, pediatricians often face challenges in accurately identifying the etiology of short stature. However, with the advancement of WES technology, several monogenic causes of growth disorders have been identified (Lui et al., 2014; Wit et al., 2016). The present study focused on single-gene mutations, and we identified the causes in 24 patients through WES. Notably, we identified a minimum of 12 genes (12/18) that affect the epiphyseal growth plate, including various aspects such as hormone signaling, paracrine factors, matrix molecules, intracellular pathways, and fundamental cellular processes (Wang et al., 2013; Wit et al., 2016). However, only one case (P65) exhibited mutations in the hormone signaling pathway. This suggests the importance of broadening the search for the causes of short stature beyond the GH signaling pathway and highlights the potential of WES in rapidly identifying the genetic causes of short stature.

The observed positive rate (24.5%) aligns with previous findings from two similar studies (135/561, 24.1%; 504/2000, 25.2%) (Yang et al., 2014; Fan et al., 2021); however, it is lower than the values observed in two cohort studies of Chinese children with short statures and suspected genetic conditions (41/114, 36.0%; 361/814, 44.3%) (Huang et al., 2018; Li et al., 2022). Upon analysis, we attribute this lower final diagnostic yield in our study to the higher proportion of patients exhibiting no additional phenotypes (68/98, 69.4%) and HtSDS between −2.5 and −3 (54/98, 55.1%) than the aforementioned studies (with no additional phenotypes: 18/114, 15.8% and 386/814, 47.4%; with HtSDS between −2.5 and −3: 37/114, 32.4% and 0/814, 0%) (Huang et al., 2018; Li et al., 2022). Hence, the severity of short stature and presence of one or more additional phenotypes increases the likelihood of a monogenic cause, warranting genetic testing.

Statistical analyses revealed four phenotypes of statistical significance, with skeletal abnormalities, facial dysmorphism, and DD/ID emerging as the most prevalent conditions. In our cohort, 12 patients were diagnosed with skeletal dysplasia, and variants related to seven genes were detected in 10 (83.3%) patients. Notably, short stature accompanied by skeletal dysplasia showed an extremely high positive diagnostic yield. Similarly, short stature with facial dysmorphism was found to significantly enhance the diagnostic rate (Huang et al., 2018; Gurovich et al., 2019; Li et al., 2022). Our cohort included 19 patients with facial dysmorphism, and variants for 13 genes were detected in 16 (84.2%) patients. The positive rate of the present study (84.2%) was higher than those of the abovementioned two studies of Chinese children with short statures (17/30, 56.7% and 131/186, 70.4%) (Huang et al., 2018; Li et al., 2022). We found that many syndromes have recognizable facial features and that describing the phenotypes in a standardized manner with genome sequencing data can improve the diagnosis rate. The incidence of DD and ID is 1%–3% in normal children, and a genetic etiology is detected in approximately 50% of children with DD/ID (Han and Lee, 2020). Many syndromes have characteristic facial features accompanied by DD/ID. Our cohort included 11 patients with DD/ID, and 10 of them were also affected by facial dysmorphism. We detected variants related to eight genes in nine patients (9/10, 90.0%), where we identified three variants in the ARID1B gene and confirmed two cases of Coffin–Siris syndrome as well as one case of Rubinstein–Taybi syndrome. These three variants are previously unreported; therefore, our findings suggest that short stature combined with facial dysmorphism and DD/ID significantly increases the likelihood of a monogenic cause. Additionally, no variants were detected in four children with SGA in the present study. A recent large cohort study suggested that the utility of WES techniques in the diagnosis of SGA is limited, indicating the need for alternative approaches, such as methylation analysis (Fuke et al., 2021). Moreover, environmental factors should be considered in the case of SGA.

A comparative analysis of the effects of GH therapy across different groups reveals significant increases in GV and HtSDS overall. However, improvements in GV and HtSDS observed in the SSS group were inconsistent. Notably, a patient with microphthalmia syndromic 6 (P85) exhibited rapid increases in GV and HtSDS (GV = 10.4 cm/year, ΔHtSDS = 0.99); conversely, one patient with 3M syndrome (P13) demonstrated slower increases in GV and HtSDS (GV = 7.4 cm/year, ΔHtSDS = 0.24), indicating less effective GH therapy. In support of our findings, we found that some experts had offered rhGH/IGF-1 treatments to some patients with 3M syndrome and reported very little or even ineffective improvements (Güven and Cebeci, 2011; Clayton et al., 2012; Khachnaoui-Zaafrane et al., 2022). This disparity suggests the differential effects of GH therapy in various syndromic short statures and highlights the importance of identifying the genetic etiology through WES for guiding rhGH treatment.

Throughout the treatment, all patients maintained their BMI SDS at stable levels, indicating no significant changes during the short-term therapy. A cohort study indicated that the BMI SDS increased significantly in children with GHD after 3 years of rhGH therapy; however, children with ISS exhibited opposite results (Cohen et al., 2014). Nevertheless, another study indicated that the BMI SDS increased slightly in children with ISS after 4 years of rhGH treatment (Cohen et al., 2013). Therefore, we believe that the long-term effects of GH therapy on BMI SDS in children with short statures of different etiologies warrant further investigation.

After the first year of therapy, the IGF-1 SDS of all groups increased significantly; the increments in the CGHD and ISS groups were lower and that in the GSD group was greater. Some studies have suggested that the ΔHtSDS in children treated with rhGH is positively correlated with IGF-1 SDS but not with the rhGH dose (Cohen et al., 2013; Cohen et al., 2014; Soliman et al., 2021). Our study revealed no significant association between IGF-1 SDS and HtSDS (*r* = 0.197); this finding may be attributed to the small sample size and short treatment duration, necessitating further research.

Comparing before and after treatment with GH, the BA was not accelerated in any of the children, and no significant differences were found between the respective groups. Some studies have revealed that high doses of rhGH therapy may lead to advanced BA (Kamp et al., 2002; Ross et al., 2015), whereas another study reported accelerated BA growth following 2 or more years of treatment (Ying et al., 2018). However, in the present study, the BA remained largely unaffected during the first year of conventional and safe-dose therapy, necessitating further investigation into whether the BA catch-up growth affects the final height.

## Conclusion

In summary, our study indicates the practicality of using WES techniques to diagnose short stature. A total of 24 (24.5%) out of 98 Chinese children with undiagnosed short statures were identified based on genetic variants using WES. The severity of the short stature and presence of one or more additional phenotypes increase the likelihood of a monogenic cause, suggesting that these phenotypes may help guide genetic testing for the etiology of the short stature. Regarding GH treatment, most children with short statures showed significant increases in HtSDS when treated for 1 year. However, we found that rhGH therapy may not be applicable for children with some syndromic short statures, which indicates that identifying the genetic etiologies of short stature by WES may have guidance value during rhGH treatment. However, more samples must be studied to further validate these findings.

## Data Availability

The causal variants were submitted to ClinVar (https:/www.ncbi.nlm.nih.gov/clinvar/) under the following accession numbers: SUB14471733; SCV005045294-SCV005045324.

## Abbreviations

NA, not available; GH, growth hormone; CGHD, complete growth hormone deficiency; IGF-1, insulin-like growth factor 1; MRI, magnetic resonance imaging; SGA, small for gestational age; DD/ID, developmental delay/intellectual disability. The *p*-values were calculated for the differences in diagnostic categories (solved or unsolved) between patients with and without a certain phenotype.

## References

[B1] Butler M. G. Miller J. L. Forster J. L. (2019). Prader–Willi syndrome—clinical genetics, diagnosis and treatment approaches: an update. Curr. Pediatr. Rev. 15, 207–244. 10.2174/1573396315666190716120925 31333129 PMC7040524

[B2] Clayton P. E. Hanson D. Magee L. Murray P. G. Saunders E. Abu Amero S. N. (2012). Exploring the spectrum of 3-M syndrome, a primordial short stature disorder of disrupted ubiquitination. Clin. Endocrinol. 77 (3), 335–342. 10.1111/j.1365-2265.2012.04428.x 22624670

[B3] Cohen L. E. (2014). Idiopathic short stature: a clinical review. JAMA 311 (17), 1787–1796. 10.1001/jama.2014.3970 24794372

[B4] Cohen P. Rogol A. D. Weng W. Kappelgaard A. M. Rosenfeld R. G. Germak J. (2013). Efficacy of IGF-based growth hormone (GH) dosing in nonGH-deficient (nonGHD) short stature children with low IGF-I is not related to basal IGF-I levels. Clin. Endocrinol. 78 (3), 405–414. 10.1111/cen.12014 22924571

[B5] Cohen P. Weng W. Rogol A. D. Rosenfeld R. G. Kappelgaard A. M. Germak J. (2014). Dose-sparing and safety-enhancing effects of an IGF-I-based dosing regimen in short children treated with growth hormone in a 2-year randomized controlled trial: therapeutic and pharmacoeconomic considerations. Clin. Endocrinol. 81 (1), 71–76. 10.1111/cen.12408 PMC4160145 24428305

[B6] Dauber A. Rosenfeld R. G. Hirschhorn J. N. (2014). Genetic evaluation of short stature. J. Clin. Endocrinol. Metab. 99 (9), 3080–3092. 10.1210/jc.2014-1506 24915122 PMC4154097

[B7] Fan X. Zhao S. Yu C. Wu D. Yan Z. Fan L. (2021). Exome sequencing reveals genetic architecture in patients with isolated or syndromic short stature. J. Genet. Genomics 48 (5), 396–402. 10.1016/j.jgg.2021.02.008 34006472

[B8] Fuke T. Nakamura A. Inoue T. Kawashima S. Hara K. I. Matsubara K. (2021). Role of imprinting disorders in short children born SGA and Silver-Russell syndrome spectrum. J. Clin. Endocrinol. Metab. 106 (3), 802–813. 10.1210/clinem/dgaa856 33236057 PMC7947753

[B9] Gravholt C. H. Viuf M. H. Brun S. Stochholm K. Andersen N. H. (2019). Turner syndrome: mechanisms and management. Nat. Rev. Endocrinol. 15, 601–614. 10.1038/s41574-019-0224-4 31213699

[B10] Guo M. H. Shen Y. Walvoord E. C. Miller T. C. Moon J. E. Hirschhorn J. N. (2014). Whole exome sequencing to identify genetic causes of short stature. Horm. Res. Paediatr. 82 (1), 44–52. 10.1159/000360857 24970356 PMC4130218

[B11] Gurovich Y. Hanani Y. Bar O. Nadav G. Fleischer N. Gelbman D. (2019). Identifying facial phenotypes of genetic disorders using deep learning. Nat. Med. 25 (1), 60–64. 10.1038/s41591-018-0279-0 30617323

[B12] Güven A. Cebeci A. N. (2011). 3M syndrome: a report of four cases in two families. J. Clin. Res. Pediatr. Endocrinol. 3 (3), 154–159. 10.4274/jcrpe.v3i3.30 21911330 PMC3184518

[B13] Han J. Y. Lee I. G. (2020). Genetic tests by next-generation sequencing in children with developmental delay and/or intellectual disability. Clin. Exp. Pediatr. 63 (6), 195–202. 10.3345/kjp.2019.00808 32024334 PMC7303420

[B14] Hauer N. N. Popp B. Schoeller E. Schuhmann S. Heath K. E. Hisado-Oliva A. (2018). Clinical relevance of systematic phenotyping and exome sequencing in patients with short stature. Genet. Med. 20 (6), 630–638. 10.1038/gim.2017.159 29758562 PMC5993671

[B15] Huang Z. Sun Y. Fan Y. Wang L. Liu H. Gong Z. (2018). Genetic evaluation of 114 Chinese short stature children in the next generation era: a single center study. Cell Physiol. Biochem. 49, 295–305. 10.1159/000492879 30138938

[B16] Juul A. Dalgaard P. Blum W. F. Bang P. Hall K. Michaelsen K. F. (1995). Serum levels of insulin-like growth factor(IGF)-binding protein-3 (IGFBP-3) in healthy infants, children, and adolescents: the relation to IGF-I, IGF-II, IGFBP-1, IGFBP-2, age, sex, body mass index, and pubertal maturation. J. Clin. Endocrinol. Metab. 80 (8), 2534–2542. 10.1210/jcem.80.8.7543116 7543116

[B17] Kamp G. A. Waelkens J. J. de Muinck Keizer-Schrama S. M. Delemarre-Van de Waal H. A. Verhoeven-Wind L. Zwinderman A. H. (2002). High dose growth hormone treatment induces acceleration of skeletal maturation and an earlier onset of puberty in children with idiopathic short stature. Arch. Dis. Child. 87 (3), 215–220. 10.1136/adc.87.3.215 12193430 PMC1719235

[B18] Khachnaoui-Zaafrane K. Ouertani I. Zanati A. Kandara H. Maazoul F. Mrad R. (2022). 3M syndrome: a Tunisian seven-cases series. Eur. J. Med. Genet. 65 (3), 104448. 10.1016/j.ejmg.2022.104448 35150935

[B19] Li H. Ji C. Y. Zong X. N. Zhang Y. Q. (2009). Height and weight standardized growth charts for Chinese children and adolescents aged 0 to 18 years. Zhonghua Er Ke Za Zhi 47 (7), 487–492. 10.3760/cma.j.issn.0578-1310.2009.07.003 19951507

[B20] Li X. Yao R. Chang G. Li Q. Song C. Li N. (2022). Clinical profiles and genetic spectra of 814 Chinese children with short stature. J. Clin. Endocrinol. Metab. 107 (4), 972–985. 10.1210/clinem/dgab863 34850017 PMC8947318

[B21] Lui J. C. Nilsson O. Baron J. (2014). Recent research on the growth plate: recent insights into the regulation of the growth plate. J. Mol. Endocrinol. 53 (1), T1–T9. 10.1530/JME-14-0022 24740736 PMC4133284

[B22] Noonan J. A. Kappelgaard A. M. (2015). The efficacy and safety of growth hormone therapy in children with noonan syndrome: a review of the evidence. Horm. Res. Paediatr. 83 (3), 157–166. 10.1159/000369012 25503994

[B23] Pedicelli S. Peschiaroli E. Violi E. Cianfarani S. Cianfarani S. (2009). Controversies in the definition and treatment of idiopathic short stature (ISS). J. Clin. Res. Pediatr. Endocrinol. 1 (3), 105–115. 10.4008/jcrpe.v1i3.53 21274395 PMC3005647

[B24] Richards S. Aziz N. Bale S. Bick D. Das S. Gastier-Foster J. (2015). Standards and guidelines for the interpretation of sequence variants: a joint consensus recommendation of the American College of medical genetics and genomics and the association for molecular pathology. Genet. Med. 17 (5), 405–424. 10.1038/gim.2015.30 25741868 PMC4544753

[B25] Ross J. L. Lee P. A. Gut R. Germak J. (2015). Attaining genetic height potential: analysis of height outcomes from the answer program in children treated with growth hormone over 5 years. Growth Horm. IGF Res. 25 (6), 286–293. 10.1016/j.ghir.2015.08.006 26363846

[B26] Siegel P. T. Clopper R. Stabler B. (1991). Psychological impact of significantly short stature. Acta Paediatr. Scand. Suppl. 377, 14–18. ; discussion 19. 10.1111/apa.1991.80.s377.14 1785309

[B27] Soliman A. Rogol A. D. Elsiddig S. Khalil A. Alaaraj N. Alyafie F. (2021). Growth response to growth hormone (GH) treatment in children with GH deficiency (GHD) and those with idiopathic short stature (ISS) based on their pretreatment insulin-like growth factor 1 (IGFI) levels and at diagnosis and IGFI increment on treatment. J. Pediatr. Endocrinol. Metab. 34 (10), 1263–1271. 10.1515/jpem-2021-0389 34291621

[B28] Wang S. R. Carmichael H. Andrew S. F. Miller T. C. Moon J. E. Derr M. A. (2013). Largescale pooled next-generation sequencing of 1077 genes to identify genetic causes of short stature. J. Clin. Endocrinol. Metab. 98 (8), E1428–E1437. 10.1210/jc.2013-1534 23771920 PMC3733853

[B29] Wit J. M. Oostdijk W. Losekoot M. van Duyvenvoorde H. A. Ruivenkamp C. A. Kant S. G. (2016). MECHANISMS IN ENDOCRINOLOGY: novel genetic causes of short stature. Eur. J. Endocrinol. 174 (4), R145–R173. 10.1530/EJE-15-0937 26578640

[B30] Wood A. R. Esko T. Yang J. Vedantam S. Pers T. H. Gustafsson S. (2009). Defining the role of common variation in the genomic and biological architecture of adult human height. Nat. Genet. 46, 1173–1186. 10.1038/ng.3097 PMC4250049 25282103

[B31] Xu S. Gu X. Pan H. Zhu H. Gong F. Li Y. (2010). Reference ranges for serum IGF-1 and IGFBP-3 levels in Chinese children during childhood and adolescence. Endocr. J. 57 (3), 221–228. 10.1507/endocrj.k09e-200 20051649

[B32] Yang Y. Muzny D. M. Xia F. Niu Z. Person R. Ding Y. (2014). Molecular findings among patients referred for clinical whole-exome sequencing. Jama 312, 1870–1879. 10.1001/jama.2014.14601 25326635 PMC4326249

[B33] Ying Y. Q. Hou L. Liang Y. Wu W. Luo X. P. (2018). Efficacy and safety of recombinant human growth hormone in treating Chinese children with idiopathic short stature. Growth Horm. IGF Res. 42-43, 80–85. 10.1016/j.ghir.2018.09.003 30343148

