# Supplementary material for: Clinical profiles and molecular genetic analyses of 98 Chinese children with short statures
Source: Front Genet. 2024 Jun 12;15:1364441. doi: 10.3389/fgene.2024.1364441 (PMC11199712; doi:10.3389/fgene.2024.1364441)
Supplement: Supplementary file 1 [file DataSheet1.doc]

**Supplementary Table 1.**The clinical characteristics of patients with monogenic short stature.Abbreviations: DD/ID,developmental delay/intellectual disability.

| **Patient** | **Gender** | **Age**  **(year)** | **Disease** | **Height**  **(SDS)** | **Other Phenotype** |
| --- | --- | --- | --- | --- | --- |
| P4 | female | 4.5 | Spondylocarpotarsal Synostosis syndrome | -2.93 | Skeletal abnormalities |
| P7 | female | 8 | Gitelman syndrome | -2.52 | Hypokalemia |
| P8 | male | 5.08 | Hypophosphatasia, infantile | -2.95 | Skeletal abnormalities |
| P9 | female | 5.5 | Rubinstein-Taybi syndrome | -3.6 | Facial dysmorphism,microcephaly,  DD/ID,broad great toes,Recurrent infections |
| P11 | female | 9.67 | Orofaciodigtal syndrome | -3.32 | Facial dysmorphism,DD/ID,Skeletal abnormalities |
| P13 | male | 2.33 | 3M syndrome 1 | -5.61 | Facial dysmorphism,Short fifth fingers |
| P16 | female | 4.92 | [Mucopolysaccharidosis type ⅢC](https://www.ncbi.nlm.nih.gov/medgen/C0086649/) | -2.55 | Facial dysmorphism,Skeletal abnormalities hepatomegaly, splenomegaly, DD/ID, Frequent respiratory infections |
| P17 | male | 4.08 | Spondylocarpotarsal Synostosis syndrome | -2.54 | Skeletal abnormalities |
| P23 | female | 3.83 | Osteogenesis imperfecta,type IV | -3.53 | Blue sclerae |
| P30 | male | 4.08 | Ritscher-Schinzel syndrome | -2.72 | Facial dysmorphism,DD/ID,ventricular septal defect |
| P38 | female | 12.17 | Noonan syndrome 3 | -4.57 | Facial dysmorphism,DD/ID,macrocephaly,  ventricular septal defect |
| P40 | female | 10.42 | Neurofibromatosis-Noonan syndrome | -3.43 | Facial dysmorphism,DD/ID,  Cafe-au-lait spots |
| P57 | female | 4 | Neurofibromatosis, type 1 | -2.85 | Cafe-au-lait spots |
| P62 | male | 4 | Wiedemann-Steiner syndrome | -2.59 | Facial dysmorphism,DD/ID |
| P65 | female | 8.42 | Growth hormone deficiency, isolated,type II | -3.02 | / |
| P66 | male | 14 | Pseudoachondroplasia | -2.56 | Skeletal abnormalities |
| P79 | male | 4.42 | Achondroplasia | -5.43 | Facial dysmorphism,Skeletal abnormalities |
| P81 | male | 8.67 | Neurofibromatosis-Noonan syndrome | -3.00 | Facial dysmorphism,Cafe-au-lait spots,macrocephaly, |
| P84 | male | 6.67 | Acromicric dysplasia | -2.96 | Facial dysmorphism,Skeletal abnormalities |
| P85 | male | 9.67 | Microphthalmia, syndromic 6 | -2.62 | Facial dysmorphism,Microphthalmia,  hearing impairment, amblyopia, inguinal hernia, equinus |
| P86 | male | 6.17 | Coffin-siris syndrome | -3.72 | Facial dysmorphism,microcephaly, DD/ID,atrial septal defect, feeding difficulties |
| P96 | male | 4.67 | Hypochondroplasia | -4.67 | Facial dysmorphism,Skeletal abnormalities |
| P97 | female | 2.25 | Hypochondroplasia | -3.42 | Facial dysmorphism,Skeletal abnormalities |
| P98 | female | 2 | Coffin-siris syndrome | -3.59 | Facial dysmorphism,microcephaly, DD/ID,atrial septal defect,absent fifth finger nail.,feeding difficulties |

**Supplementary Table 2:The Evidence 0f the American College of Medical Genetics and Genomics(ACMG) guidelines.**

| Gene | position | Sequencing variants (hg19) | Evidence of ACMG | Classification | Patient |
| --- | --- | --- | --- | --- | --- |
| **Autosomal dominant** | | | | | |
| *CREBBP* | Exon31 | NM_004380.2:c.6111del(p.Arg2037SerfsTer3) | PVS1_Strong+PM2_Supporting+PP4 | LP | P9 |
| *COL1A2* | Exon48 | NM_000089.3:c.3256C>T(p.Gln1086*) | PVS1+PM2_Supporting+PP4 | P | P23 |
| *KRAS* | Exon2 | NM_004985.4:c.40G>A(p.Val14Ile) | PS3+PS4_Moderate+PM1+PM2_Supporting+PP3 | P | P38 |
| *NF1* | Exon1 | NM_000267.3:c.60G>C(p. Gln20His) | PS2_Srong+PM1+PM2_Supporting | LP | P40 |
|  | Exon9 | NM_000267.3:c.943C>T(p.Gln315Ter) | PVS1+PS3+PM2_Supporting+PP4 | P | P81 |
|  | Exon7 | NM-001042492.2:c.693delT(p.Phe231fs) | PVS1+PM2_Supporting+PS2_Strong | P | P57 |
| *KMT2A* | Exon5 | NM_001197104.1:c.3460C＞T(p.Arg1154Trp) | PS3_supporting+PS4_strong+PS2_very strong+PM1+PM2_Supporting | P | P62 |
| *GH1* | Exon5 | NM-000515.4:c.626G>A(p.Arg209His) | PM1+PM2_Supporting+PP1_Strong | LP | P65 |
| *COMP* | Exon16 | NM-000095.2:c.1829A>G(p.Tyr610Cys) | PM1+PM2_Supporting+PP3+PP1+PP4 | LP | P66 |
| *FGFR3* | Exon9 | NM_000142.4:c.1138G>A(p.Gly380Arg) | PS1+PS3_moderate+PS4_Strong+PM1+PM2_Supporting | P | P79 |
|  | Exon12 | NM_000142.4:c.1619A>G(p.Asn540Ser) | PS4_Supporting+PM1+PM2_Supporting+PM5_Strong+PP3 | P | P96 |
|  | Exon12 | NM_000142.4:c.1620C>G(p.Asn540Lys) | PS4_Strong+PM1+PM2_Supporting+PM5_Strong+PP4 | P | P97 |
| *FBN1* | Exon42 | NM_000138.4:c.5183C>T(p.Ala1728Val) | PS4_Moderate+PM2_Supporting+PM5_Strong+PP3+PP4 | LP | P84 |
| *BMP4* | Intron3 | NM_001202.6:c.371-2A>G | PVS1+PM2_Supporting+PP4 | LP | P85 |
| *ARID1B* | Exon20 | NM_020732.3:c.6683C>A(p.Ser2228*) | PS2+PM2_Supporting+PM4+PP4 | LP | P86 |
|  | Exon18 | NM_020732.3:c.4520delA(p.Asn1507fs) | PVS1-Strong+PS2+PM2_Supporting+PP4 | P | P98 |
| **Autosomal recessive** | | | | | |
| *FLNB* | Exon13 | NM_001457.3:c.1945C>T(p.Arg649Ter） | PVS1+PM2_Supporting+PP4 | P | P4 |
|  | Exon19 | NM_001457.3:c.2774G>A(p.Gly925Asp) | PM1+PM2_Supporting+PP3_Moderate+PP4 | LP |  |
|  | Exon16 | NM_001457.3:c.2452C>T(p.Arg818Ter) | PVS1+PM2_Supporting+PP4 | P | P17 |
|  | Exon28 | NM_001457.3:c.4819C>T(p.Arg1607Ter） | PVS1+PM2_Supporting+PM3+PP4 | P |  |
| *SLC12A3* | Exon1 | NM_000339.2:c.179C>T(p.Thr60Met) | PS3+PS4+PM2_Supporting+PP3+PP4 | P | P7 |
|  | Exon10 | NM_000339.2:c.1316G>T( p.Gly439Val) | PM2_Supporting+PM3+PM5+PP3+PP4 | LP |  |
| *ALPL* | Exon4 | NM_000478.4:c.212G>A(p.Arg71His) | PS4_Strong+PM1+PM2_Supporting+PM3+PP4 | P | P8 |
|  | Exon6 | NM_000478.4: c.571G>A(p.Glu191Lys) | PS3+PM1+PP4 | LP |  |
| *C5orf42* | Exon20 | NM_023073.3:c.3577C>T (p.Arg1193Cys) | PM1+PM2_Supporting+PM3_Strong | LP | P11 |
|  | Exon20 | NM_023073.3:c.3599C>T (p.Ala1200Val) | PS4+PM1+PM2_Supporting+PM3+PM5 | P |  |
| *CUL7* | Exon23 | NM_014780.4:c.4318C>T(p.Arg1440*) | PVS1+PM2_Supporting+PP4 | P | P13 |
|  | Exon15 | NM_014780.4:c.2875dupA(p.Thr959fs) | PVS1+PM2_Supporting+PM3+PP4 | P |  |
| *HGSNAT* | Exon15 | NM_152419.2:c.1516C>T(p.Arg506Ter) homozygous | PVS1+PM2_Supporting+PP4 | P | P16 |
| *KIAA0196* | Exon3 | NM_014846.3:c.232C>T(p.Gln78Ter) | PVS1+PM2_Supporting+PP4 | P | P30 |
|  | Exon20 | NM_014846.3:c.2489G>A(p.Arg830Gln) | PM1+PM2_Supporting+PM3_Strong+PP4 | LP |  |
